# Cold Expansion Process with Multiple Balls—Numerical Simulation and Comparison with Single Ball and Tapered Mandrels

**DOI:** 10.3390/ma13235536

**Published:** 2020-12-04

**Authors:** David Curto-Cárdenas, Jose Calaf-Chica, Pedro Miguel Bravo Díez, Mónica Preciado Calzada, Maria-Jose Garcia-Tarrago

**Affiliations:** 1 CIMa Research Team, Department of Civil Engineering, University of Burgos, Av. Cantabria s/n, 09006 Burgos, Spain; jcalaf@ubu.es (J.C.-C.); pmbravo@ubu.es (P.M.B.D.); mpreciado@ubu.es (M.P.C.); 2 Department of Electromechanical Engineering, University of Burgos, Av. Cantabria s/n, 09006 Burgos, Spain; mjgtarrago@ubu.es

**Keywords:** cold expansion, swaging, ball, aeronautical, simulation, experimental

## Abstract

Cold expansion technology is an extended method used in aeronautics to increase fatigue life of holes and hence extending inspection intervals. During the cold expansion process, a mechanical mandrel is forced to pass along the hole generating compressive residual hoop stresses. The most widely accepted geometry for this mandrel is the tapered one and simpler options like balls have generally been rejected based on the non-conforming residual hoop stresses derived from their use. In this investigation a novelty process using multiple balls with incremental interference, instead of a single one, was simulated. Experimental tests were performed to validate the finite element method (FEM) models and residual hoop stresses from multiple balls simulation were compared with one ball and tapered mandrel simulations. Results showed that the use of three incremental balls significantly reduced the magnitude of non-conforming residual hoop stresses and the extension of these detrimental zone.

## 1. Introduction

The need to increase the lifetime of critical holes in aeronautical metallic structures, reducing the stress level of fluctuating tensile loads, resulted in investigations focused on relieving the adverse effect of making holes in sheet plates. Besides the stress concentration, a hole implies new surfaces where cracks are initiated [1,2].

The main joining process used in aircrafts implies making holes even in highly loaded zones. Some fatal accidents involving the known problem of fatigue pushed to aircraft manufacturers to investigate methods to increase fatigue life. In 1974, Boeing’s material research and development department at the Boeing Commercial Airplane Company in Seattle published an article introducing the concept of ‘sleeve coldworking’ applied to fasteners holes [3,4]. A tapered mandrel was pulled through a slightly smaller hole. A region around the hole was yielded and the remaining elastic zone generated residual compressive stresses.

The first records of this phenomenon are dated from the 19th century in the artillery industry. Gun barrels showed a significant enhance in their service life limit when interference hoops were installed. In the 1920s, it was noticed that gun barrels that were triggered once after the manufacturing process with an oversized cannonball—to test the capability of the gun—had higher endurance. Although manufacturers do not know the cause for this improvement, they were introducing compressive residual stresses in the barrel structure. Searching for higher nominal pressures in vessels and the use of new stronger materials during the Second Industrial Revolution motivated new methods to increase these compressive residual stresses.

Different industrial sectors developed a range of processes for the generation of residual stresses. Autofrettage of thick-walled cylinders has its industrial applications (e.g., gun barrels or high pressure vessels) and it has been deeply studied [5,6,7,8]. In the specific case of the aerospace industry, the focus was centered on fasteners and bolts holes, where Boeing Company developed the first application of the mechanical autofrettage in aircraft airframes: the cold expansion process. In this manufacturing process, a mechanical gadget (the mandrel) is forced to pass along the hole at room temperature [9]. In this specific investigation, different mandrel geometries (ball and tapered ones) were compared.

There are other methodologies and industrial processes focused on generation of compressive residual stresses, such as shot peening [10], laser shock peening [11] or ultrasonic peening [12]. The main purpose was the same—yielding the material at the surface and forcing the internal layers to recover their original position, generating compressive stresses in the yielded section. But these alternative methodologies are not focused on small holes.

**Figure 1:**
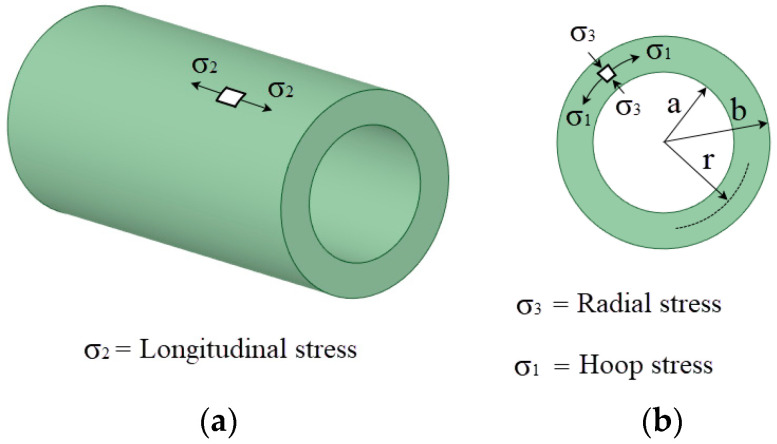
Sketch of stress field nomenclature (**a**) Longitudinal; (**b**) Radial and hoop.

Polar coordinates are typically used to represent the residual stresses generated after the cold expansion process: hoop, radial and longitudinal as shown in Figure 1.

The stress component that best prevents the initiation and the propagation of cracks is the compressive stress in hoop direction and this research was focused on this component.

In the cold expansion process, residual compressive and tensile stresses are generated along the radial direction of the hole. The location and the magnitude of these stresses are the key to obtain an effective cold expansion. Different parameters affect to this stress distribution: mandrel-hole interference level, friction coefficients, material mechanical properties—yield strength and strain-hardening—elastic remaining zone after the process—holes in aerostructures are usually situated in the middle of wide sheets—geometry of the mandrel—this investigation analyzed balls and tapered mandrels and so forth.

For the specific case of the mandrel geometry, there are a wide published research focused on the tapered models [13,14,15,16] but limited investigations on ball geometries [17,18]. The motivation behind the use of ball geometries, as an alternative to tapered mandrels, comes from its consolidated manufacturing process based on their use for ball bearings industry. Balls are cheaper and they have extremely fine tolerances and significant smooth surface. Baltach [19] simulated both tapered and ball mandrels in cold-expansion processes. He concluded that ball geometry generated non-conforming hoop stresses at the inlet and outlet regions of the hole, in comparison with tapered mandrels, which generated better results. Additionally, low surface roughness, obtained after the cold expansion process, significantly delays the crack initiation process [20,21].

This investigation analyzed an alternative cold-expansion process in order to improve the residual stress field obtained with the ball geometry. The novelty of this research was focused on the way to reach the total interference of mandrel-hole interaction: multiple substeps with balls with an incremental interference were applied instead of a single cold-expansion step, obtaining a significant improvement in the residual stresses at inlet region of the hole.

According to References [16,22,23,24] mandrel-hole interference level is established from 1% or less up to 5–6%. This investigation considered that interference levels around 0.5–1.0% were high enough considering the shape of the residual hoop stresses.

## 2. Materials and Methods

**Figure 2:**
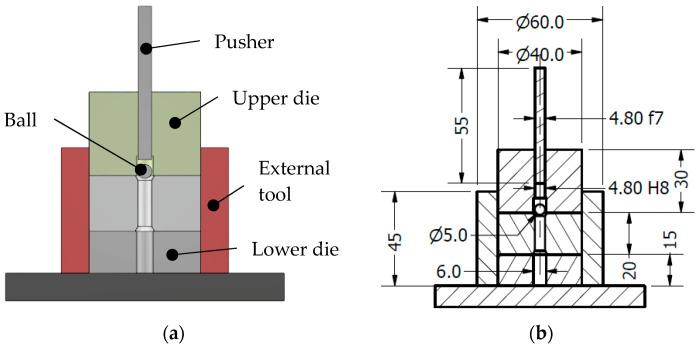
(**a**) Test set-up; (**b**) General geometry definition.

**Figure 3:**
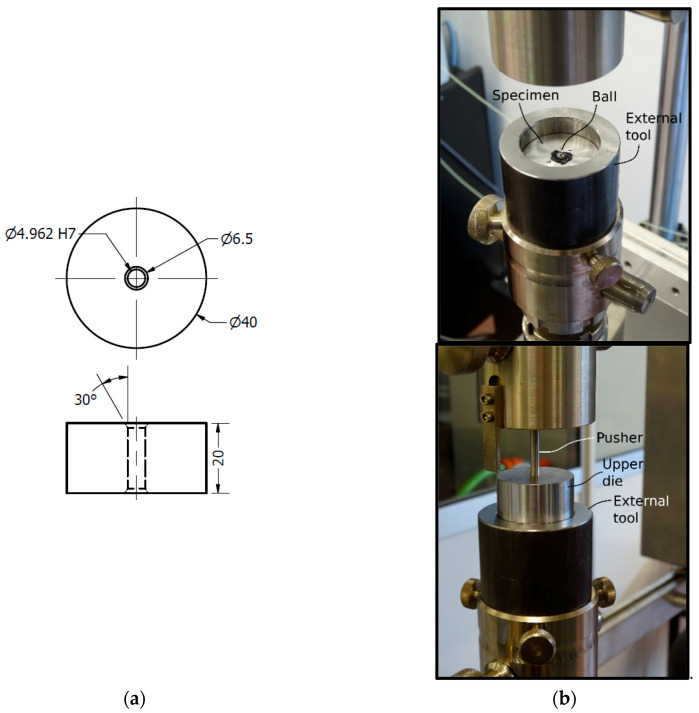
(**a**) Specimen geometry definition and (**b**) test set-up.

Experimental tests were performed in order to validate the later finite element method (FEM) simulations. The set-up and geometry definition of each part is shown in Figure 2 and Figure 3. The tested specimen consisted of a cylinder with a diameter of 40 mm and a height of 20 mm with an internal hole of 4.962 H7 mm. This hole was drilled with the next manufacturing sequence: drill bit of Ø4.90 mm and three reamer steps of Ø4.935 mm, Ø4.953 mm and Ø4.962 mm. This manufacturing drilling process is a standard in hole drilling in aeronautics. Chamfers at both holes’ ends were machined to avoid misalignments during the cold-expansion process. A 5.000 mm lubricated ball with precision grade G10 (ISO 3290) was forced to pass through the hole with a pusher of 4.8 mm diameter. The pusher displacement was guided with an upper die placed over the specimen. A lower die was fitted to ensure that the ball had enough room to end the test undisturbed. All the parts were aligned with an external tool with outer and inner diameters of 60 mm and 40 mm respectively and they were lubricated with Molykote BR2-PLUS^®^ grease to reduce metal-metal friction between different parts. During the test, the pusher load versus the pusher displacement were measured and registered. Cold expansion process was performed in a Zwick/Roell KAPPA 050 DS testing machine with a load cell Xforce P with a nominal force of 5kN. The pusher displacement was registered with crosshead signals (resolution: 0.1 µm). Contributions of crosshead deformation in the registered displacement was non-significant for the measured load range during the test.

**Figure 4:**
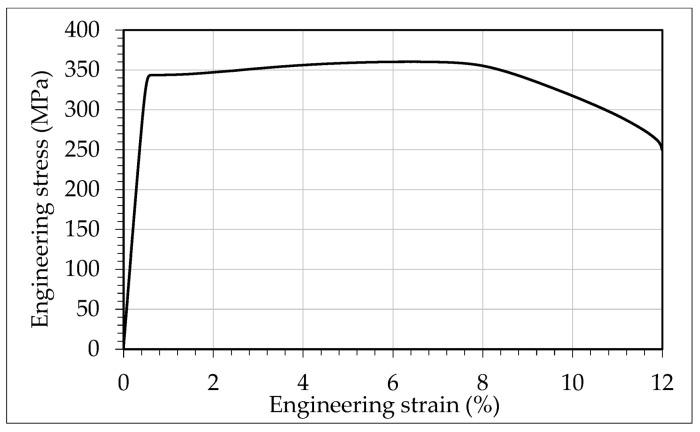
Engineering stress-strain curve for Al6082 T6.

The selected materials for each part and their mechanical properties are shown in Table 1. A tensile test specimen was machined from the same raw material of the drilled cylindrical specimen and was tested according to ISO 6892-1. Figure 4 shows the registered engineering stress-strain curve.

The simulations were performed with ANSYS v18.2 software. In order to reduce the computational costs, the FEM models were simplified with axisymmetric simulations. The plastic behavior of Al6082 T6 was simulated with a Chaboche’s kinematic model with one backstress (see Equation (1)). In order to obtain the coefficients of the Chaboche’s model, a non-linear least squares regression method was applied to the previously obtained experimental true stress-strain curve (see Table 2).

(1) $$\sigma \;=\;\frac{C_{1}}{\gamma_{1}}\left(1-e^{-\gamma_{1}\varepsilon_{p}}\right).$$

**Figure 5:**
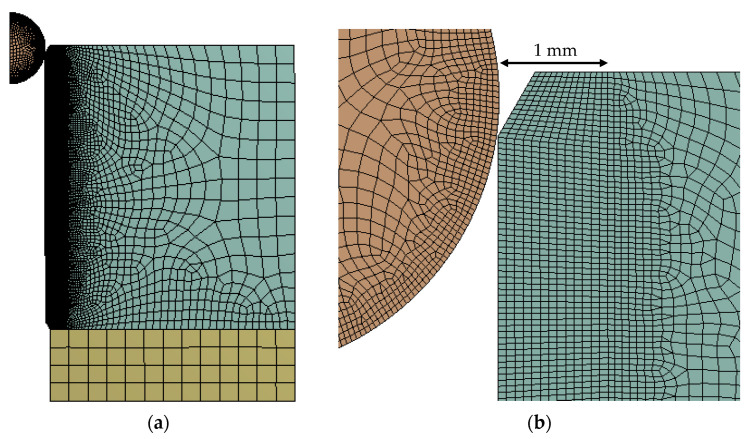
(**a**) Finite element method (FEM) model of the cold-expansion process with one ball; (**b**) Detail of the mesh size at the inner radius.

**Figure 6:**
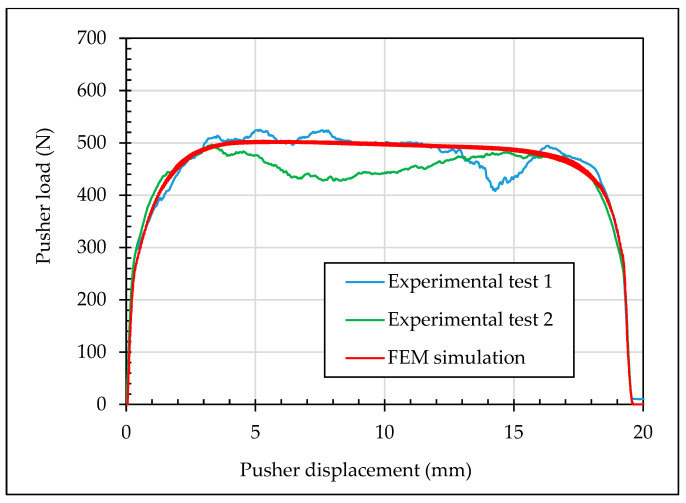
Experimental tests and FEM simulation of the cold-expansion process.

First, a validation of the FEM models was performed with a simulation of the experimental cold-expansion test. During the design of this FEM model, there were two unknown variables: the frictional coefficient *µ* of the interaction between the ball and the specimen and the real hole diameter *d* of the specimen. Although both ones affected to the maximum pushing load of the ball, the shape of the experimental load-displacement curve was achieved with a specific combination of a frictional coefficient *µ* = 0.061 and a hole diameter *d* = 4.974 mm within the tolerances 4.962H7 of the hole. During this FEM analysis, a mesh sensibility check was performed. It was considered fine regular mesh of 0.05 mm in the first 1 mm in radial direction, where yielding took place during the simulation of the cold expansion process. Beyond this 1 mm, stress gradient was considerably lower and mesh size was increased in concordance. Same 0.05 mm mesh size was simulated in the outer diameter of the ball (see Figure 5). Figure 6 shows the comparison between the FEM simulation and the two experimental trials. In order to quantify the deviation of the simulation, the Root Mean Squared Error (RMSE) between the experimental trials and the FEM simulation was calculated with the Equation (2), obtaining: *RMSE_exp_*_1_ = 21.9 N and *RMSE_exp_*_2_ = 35.8 N.

(2) $$RMSE\;=\;\sqrt{\frac{\sum_{i\;=\;1}^{n\;}{\left(s_{i}-e_{i}\right)}^{2}}{n}},$$

where, *s_i_* is the load estimated by the simulation for the *i* displacement, *e_i_* is the load registered by the experimental test for the *i* displacement and *n* is the number of positions considered for the analysis of the *RMSE*.

**Figure 7:**
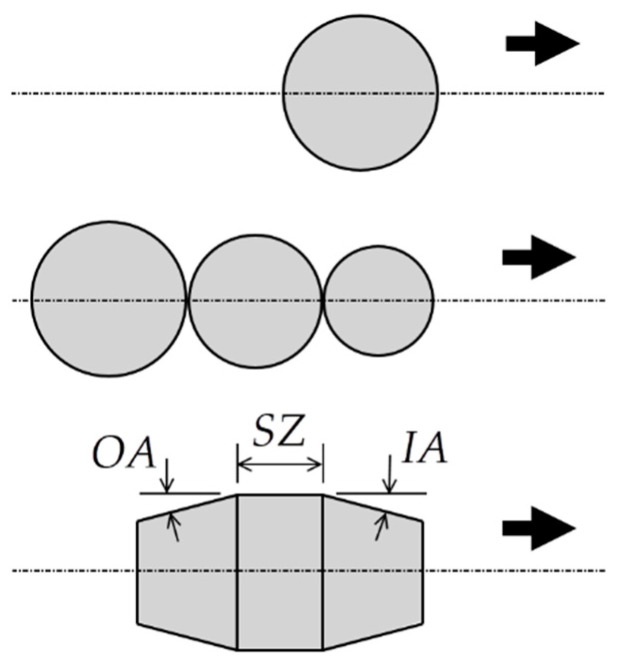
Geometrical parameters of mandrel geometries: outlet angle (OA), straight zone (*SZ*) and inlet angle (IA).

**Figure 8:**
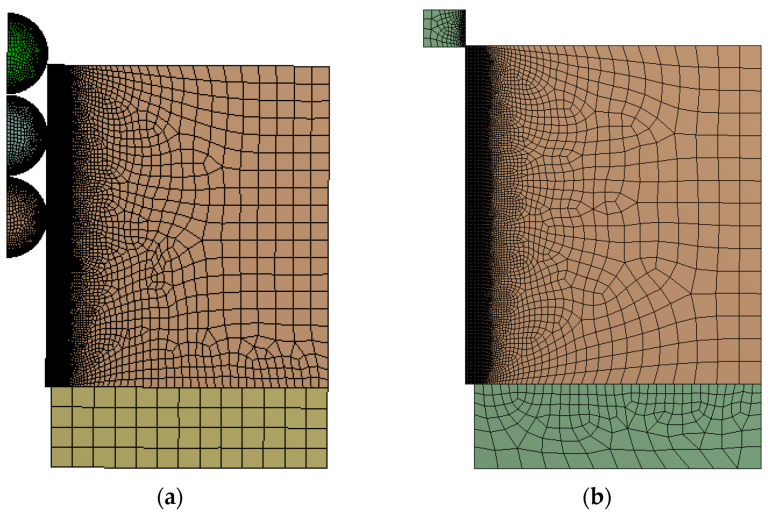
FEM models of the cold-expansion process with: (**a**) three balls and (**b**) tapered mandrel.

The second part of this investigation involved FEM simulations based on the previously validated model. A similar hole diameter of 4.974 mm and different gadgets (mandrel and balls) were used. Three simulations were performed and compared (see Figure 7):

- One ball with 5.000 mm diameter.
- Three balls with 4.980, 4.990 and 5.000 mm diameters (see Figure 8a). In this simulation, each ball was moved beside the next with three different enforced displacements. Thus, no contact was established between balls.
- One optimized tapered mandrel with an outer diameter of 5.000 mm (see Figure 8b).

**Figure 9:**
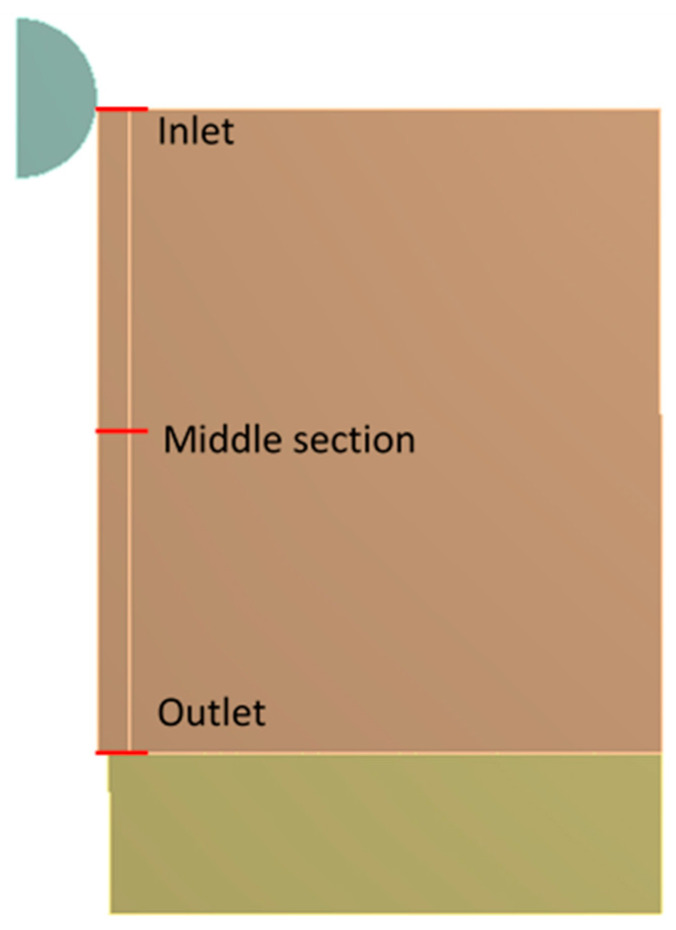
Paths for registering of the residual hoop stresses of each simulation.

From each simulation, residual hoop stresses along the radial direction were obtained for the inlet zone, middle section and outlet zone of the hole (see Figure 9).

Calaf-Chica et al. [13] showed the significance of selecting the optimal geometry for tapered mandrels. Three parameters were targeted: length of straight zone (*SZ*), inlet angle (*IA*) and outlet angle (*OA*) (see Figure 7). Outlet angle has not high influence and is generally matched with the inlet angle.

**Figure 10:**
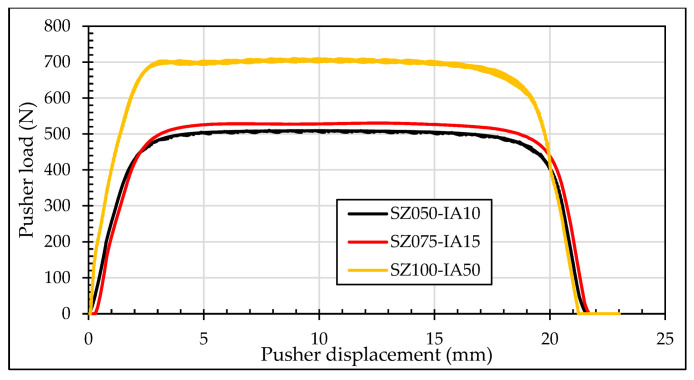
Pushing loads for tapered mandrel simulations.

An iterative process was performed to determine the best tapered mandrel geometry in order to obtain the highest residual compressive hoop stresses at inlet and outlet zones after the cold expansion process. This calculation was executed considering a similar level of pushing loads needed for the process (comparing with cold expansion performed with a single ball geometry; around a maximum load of 500 N). From Reference [13], it was deduced that an increment of the length of the straight zone or a reduction of the inlet angle increase the pushing load. In this iterative process, three different *SZ*s were evaluated (*SZ* = {0.50, 0.75, 1.00} mm) obtaining the appropriate *IA* to reach the most similar pushing load (*IA* = {1.0, 1.5, 5.0}°). Figure 8b shows the FEM model used for this iterative process. Figure 10 represents the pushing load for the three evaluated cases, where case with *SZ* = 1.00 mm showed extreme loads unable to be reduced by increments of the *IA*. Thus, in order to compare cold expansion processes with similar pushing loads, the *SZ* should not be higher than 1.00 mm.

**Figure 11:**
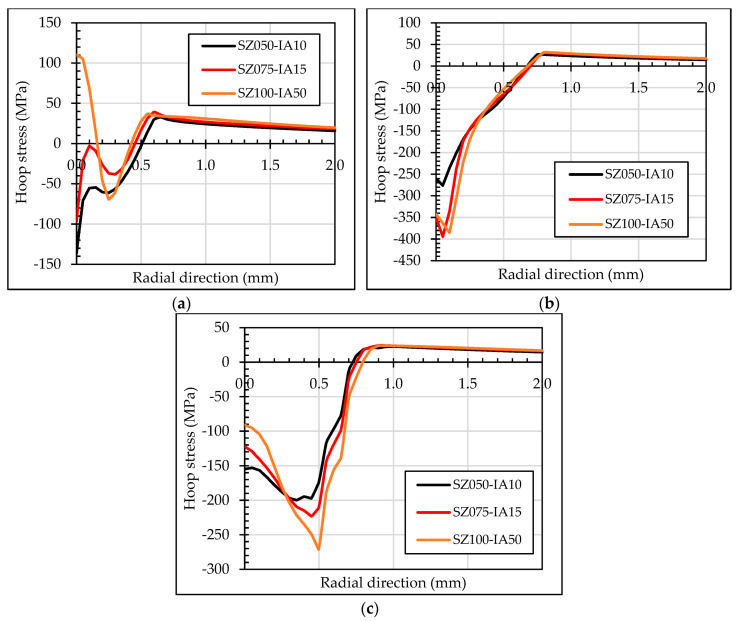
Residual hoop stresses in radial section at (**a**) inlet zone; (**b**) middle section; (**c**) outlet zone for tapered mandrel geometry evaluation.

Figure 11 shows the residual hoop stresses along the radial direction for the three evaluated set-ups for tapered mandrel geometry. Although the most compressive residual hoop stress at the inlet zone was obtained for the case SZ050-IA10, it showed a significant reduction of the residual hoop stresses at the middle section. As consequence, the geometry with *IA* = 1.5° and *SZ* = 0.75 mm was selected as the optimum for the comparison with the ball geometry.

## 3. Results and Discussion

**Figure 12:**
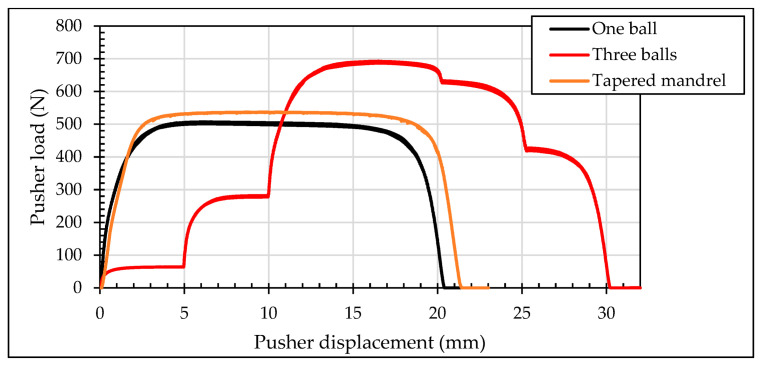
Comparison of pushing loads with different mandrels.

Figure 12 shows the pushing loads during the cold-expansion process of the three simulations with one ball, three balls and tapered mandrel. Required loads for the process with three balls were stepped as each ball contacted the specimen. Required loads for the process with tapered mandrel were intentionally delimited as described previously.

**Figure 13:**
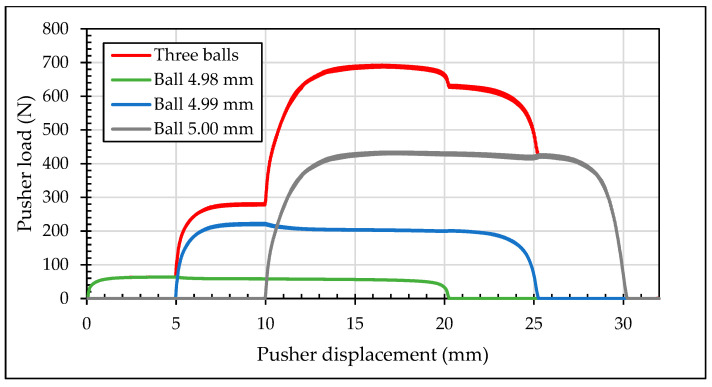
Loads decomposition for each ball in three balls test.

Meshing of the three balls case is presented in Figure 8a. In this simulation, the three balls pass, aligned through the hole, at the same time. At first, only the 4.980 mm diameter ball gets in contact with the specimen. Just after some displacement of the balls, the 4.990 mm diameter ball also gets in contact with the specimen and the necessary pushing load jumps up to a higher value. Finally, the 5.000 mm diameter ball also gets in contact with the specimen. Along some displacement, the three balls are in contact with the specimen. Figure 13 shows the pusher load component for each ball and the sum up of the three balls.

**Figure 14:**
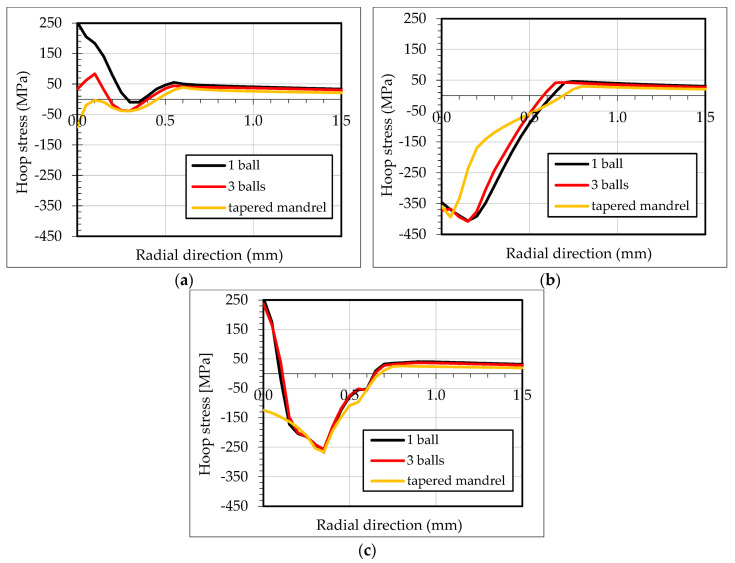
Residual hoop stresses in radial section at (**a**) inlet zone; (**b**) middle section; (**c**) outlet zone.

Figure 14 illustrates the residual hoop stresses along the first 1.5 mm in the radial direction. The first 0.3 mm of the inlet zone showed tensile residual hoop stresses for the simulation with one ball. The simulation with three balls significantly reduced the tensile residual stresses as well as the area affected to 0.2 mm. Minor variation in the compressive residual hoop stresses are shown in the middle section comparing the three simulations. The one ball and three balls FEM models caused in the outlet zone a 0.1 mm of tensile residual hoop stresses. It is noted that 0.15 mm inside the outlet zone, residual hoop stresses for the one and three balls simulations were aligned with the tapered mandrel simulation.

**Figure 15:**
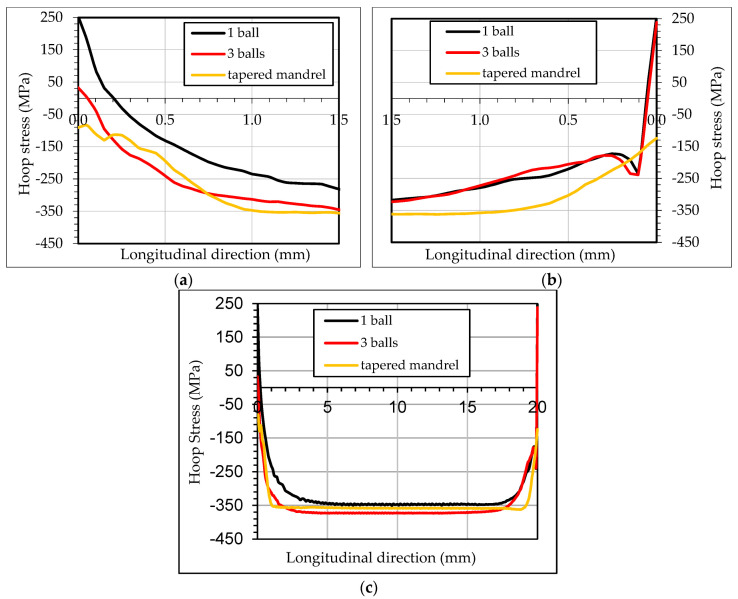
Residual hoop stresses at inner diameter along the longitudinal direction at (**a**) inlet zone; (**b**) outlet zone; (**c**) full longitudinal distance.

Figure 15 shows the distribution of residual hoop stresses along the first (Figure 15a) and the last (Figure 15b) 1.5 mm in the longitudinal direction at the hole radius. Figure 15c shows the residual hoop stress distribution along full longitudinal distance. In the first 1.5 mm, the simulation with one ball showed 0.2 mm area of tensile residual hoop stresses, while using tapered mandrel generated compressive residual hoop stresses. Simulation with three balls showed that magnitude and the affected area with tensile hoop stresses were reduced significantly compared with one ball set-up. In the last 1.5 mm, the simulation with one and three balls showed similar results with high residual tensile hoop stresses in the last 0.05 mm of the specimen, while the simulation with tapered mandrel showed again compressive residual hoop stresses.

**Figure 16:**
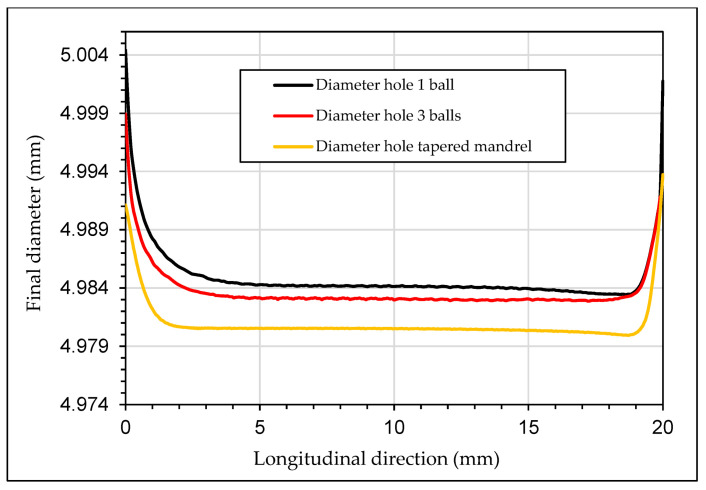
Hole diameter after the cold-expansion process.

Figure 16 shows the final hole diameter after the simulated cold-expansion processes. From the initial diameter of 4.974 mm to: 4.984 mm for one ball case, 4.983 mm for three balls case and 4.980 mm for tapered mandrel case at the middle section of the specimen. At the inlet and the outlet, the final diameters were higher in all the cases.

**Figure 17:**
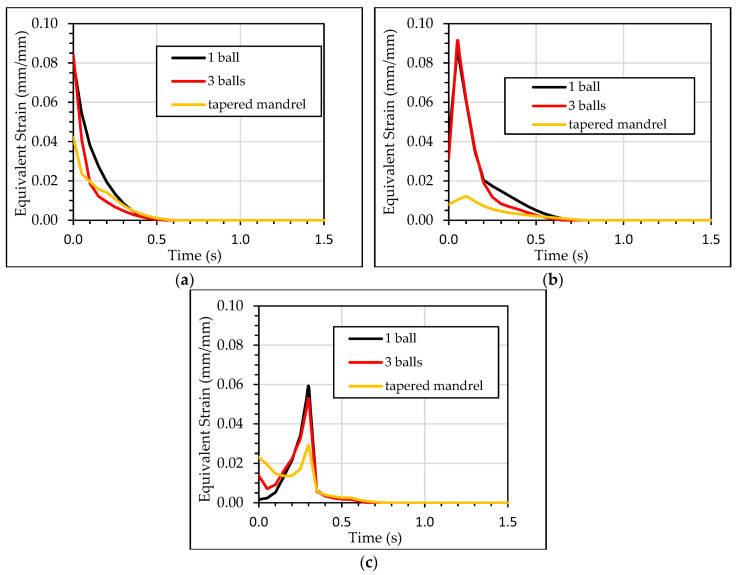
Equivalent plastic strain (**a**) inlet zone; (**b**) middle section; (**c**) outlet zone.

**Figure 18:**
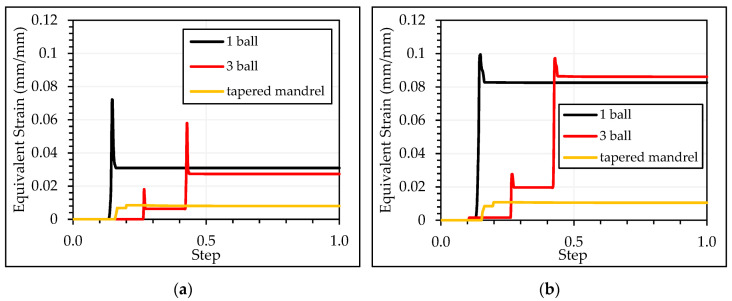
Equivalent Plastic Strain (**a**) Minimum; (**b**) Maximum for a mesh element located at the inner diameter of the hole.

Figure 17 shows the equivalent plastic strain stresses along the first 1.5 mm in the radial direction at three locations: the inlet zone, the outlet zone and the middle section. Tapered mandrel case showed lower values of the equivalent plastic strain, with a significant difference in the middle section. Figure 18 shows the evolution of the equivalent plastic strain in a mesh element in the location of the hole radius and in a section where the influence of inlet and outlet zones were neglected. When the ball or balls contacted this mesh element, the equivalent plastic strain was increased. When the ball displacement progressed, the contact between the ball and the mesh element disappeared. In that moment, the equivalent plastic strain in the mesh element was reduced. In the first of the balls in the three balls simulation, no significant plastic strain was generated. This is explained by the low interference level for this first ball. Tapered mandrel simulation showed a different behavior. For the same level of interference, the equivalent plastic strain values are very low compared with the balls’ simulations and it showed low equivalent plastic strain reduction after the mandrel contact.

The reduction of the equivalent plastic strain after the ball/hole contact can be explained by the Bauschinger effect. Yielded material at the inner diameter of the hole is deformed in hoop direction in a tensile mode during the cold expansion process. The residual hoop stresses after the cold expansion process are in the opposite sense (compressive hoop stresses). Higher level of interference generated a saturation of the compressive capability of the yielded material due to the presence of the Bauschinger effect [25] reducing the equivalent plastic strain (see Figure 18).

**Figure 19:**
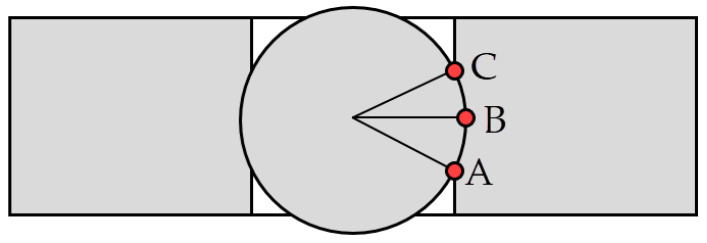
Schematic scheme for ball-hole contact in a cold-expansion process.

The initial stages of the yielding behavior during the cold expansion process with balls take place from the first contact point ‘A’ to the maximum yielding at point ‘B’ (see Figure 19). From this point ‘B’ to point ‘C,’ the interference is progressively reduced and the elastic zone of the specimen (located at higher radius) partially recovers its original radial position compressing the yielded zone that has less springback radial movement. Meanwhile, the ‘AB’ contact for tapered mandrel is a straight line with a constant angle of 1.5°.

**Figure 20:**
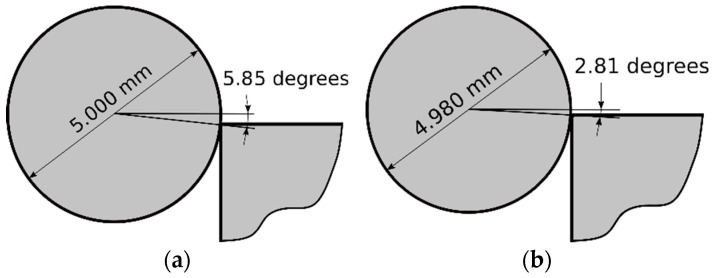
Ball-hole angle with ball diameter of (**a**) 5.000 mm; (**b**) 4.980 mm.

This investigation evaluated if this inlet angle was one of the parameters that affected to the distribution of the residual hoop stresses at the inlet zone. For the 5.000 mm diameter ball, the contact angle ranges from 5.85° to 0° (see Figure 20a) and for the 4.980 mm diameter ball, it ranges from 2.81° to 0° (see Figure 20b). Calaf-Chica et al. [13] showed the importance of the inlet angle for tapered mandrels and similar importance was expected for balls. The use of different balls’ diameters in sequential substeps would reduce the inlet contact angle.

Residual hoop stresses at inlet of the hole showed that the cold expansion process using one ball generated higher tensile residual hoop stresses at the inlet and the outlet zones of the specimen. The use of three balls, which reduced the ball-hole contact angle, significantly reduced the magnitude of tensile residual hoop stresses and the affected zone by these detrimental tensile stresses. Residual hoop stresses at outlet zone showed a different behavior. The use of multiple balls did not significantly affect to the distribution of the residual hoop stresses. Additional processes at the inlet and outlet zones [26,27] could complement the benefits of these cold expansion.

**Figure 21:**
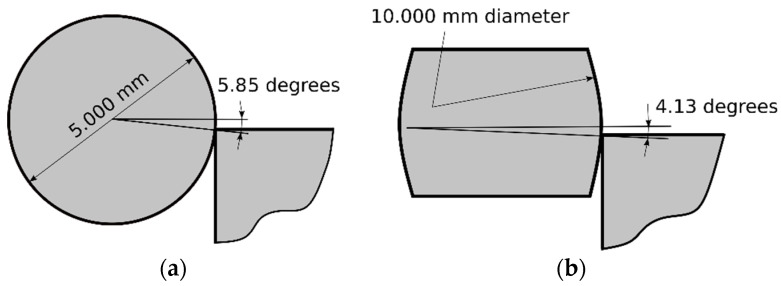
Mandrel-hole angle with contact diameters of (**a**) 5.000 mm; (**b**) 10.000 mm.

An additional FEM simulation was done in order to verify that ball-hole initial contact angle was the geometrical parameter that influenced on the residual hoop stresses at the inlet zone. A cold expansion simulation following similar set-up of previous FEM models was performed with: a ball mandrel of 5.000 mm diameter and a hole of 4.974 mm (initial contact angle of 5.85°); and a mandrel with an outer curvature of 10.000 mm diameter but with similar mandrel-hole interference (5.000 − 4.974 = 0.026 mm). The mandrel-hole initial contact angle for this geometry was equal to 4.13°. Figure 21 shows the set-up for both models.

**Figure 22:**
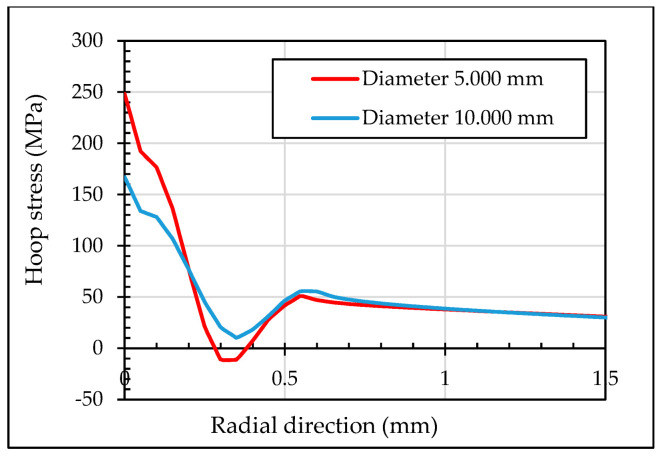
Residual hoop stresses in radial section at inlet zone.

Figure 22 shows the residual hoop stresses for both models at inlet zone in radial direction. This demonstrates that when ball-hole set-up reduces the initial inlet angle, the residual hoop stresses at inlet zone are improved.

## 4. Conclusions

In some applications, like aeronautics, cold expansion is an iterative process in some critical structural holes. After several uses, the mandrel must be replaced as a spare part. Balls are much cheaper, they show better tolerances and surface finishes and they have a more standardized manufacturing process. Reducing the adverse effect at inlet and outlet zones, balls could be considered an alternative to the tapered mandrels. Focused on this objective, this research has given rise to the following conclusions:

1. The origin of the non-conforming tensile residual hoop stresses in the inlet zone in cold expansion processes performed with a single ball, comes from the inherent inlet angle linked with the ball-hole interference.
2. The use of multiple balls with incremental interference reduces the interference level, reducing also the inlet angle. This fact brings a decrease of the non-conforming tensile residual hoop stresses at the inlet zone.
3. This novel cold expansion process with multiple balls improves the obtained residual stress field compared with the single ball method and it also approaches the results obtained with tapered mandrels.

As forthcoming research, experimental tests for this novel cold expansion process including a fatigue analysis should be performed to demonstrate and validate the conclusions obtained in this investigation.

## Figures and Tables

**Table 1:** Materials selection for each part of the experimental test.

Material	*E* (GPa)	*σ_y_* (MPa)	*ν*	Used in
Al6082 T6	70.5	343.7	0.33	Cylindrical specimen
Tungsten	600.0	-	0.20	Ball
Steel	200.0	-	0.30	Tooling

**Table 2:** Calculated coefficients for the Chaboche’s kinematic model.

*C*_1_ (MPa)	*γ*_1_	*R* ^2^
865	6.385	0.994
